# Recording the Presence of *Peanibacillus larvae larvae* Colonies on MYPGP Substrates Using a Multi-Sensor Array Based on Solid-State Gas Sensors

**DOI:** 10.3390/s21144917

**Published:** 2021-07-19

**Authors:** Beata Bąk, Jakub Wilk, Piotr Artiemjew, Jerzy Wilde

**Affiliations:** 1 Department of Poultry Science and Apiculture, Faculty of Animal Bioengineering, University of Warmia and Mazury in Olsztyn, Sloneczna 48, 10-957 Olsztyn, Poland; teofil.wilk@uwm.edu.pl (J.W.); jerzy.wilde@uwm.edu.pl (J.W.); 2 Faculty of Mathematics and Computer Science, University of Warmia and Mazury in Olsztyn, 10-719 Olsztyn, Poland; piotr.artiemjew@uwm.edu.pl

**Keywords:** *Peanibacillus larvae larvae*, MYPGP, gas sensor, electronic nose, k nearest neighbor algorithm, naïve Bayes classifier, weighted voting classifier

## Abstract

American foulbrood is a dangerous disease of bee broods found worldwide, caused by the *Paenibacillus larvae larvae* L. bacterium. In an experiment, the possibility of detecting colonies of this bacterium on MYPGP substrates (which contains yeast extract, Mueller-Hinton broth, glucose, K2HPO4, sodium pyruvate, and agar) was tested using a prototype of a multi-sensor recorder of the MCA-8 sensor signal with a matrix of six semiconductors: TGS 823, TGS 826, TGS 832, TGS 2600, TGS 2602, and TGS 2603 from Figaro. Two twin prototypes of the MCA-8 measurement device, M1 and M2, were used in the study. Each prototype was attached to two laboratory test chambers: a wooden one and a polystyrene one. For the experiment, the strain used was *P. l. larvae* ATCC 9545, ERIC I. On MYPGP medium, often used for laboratory diagnosis of American foulbrood, this bacterium produces small, transparent, smooth, and shiny colonies. Gas samples from over culture media of one- and two-day-old foulbrood *P. l. larvae* (with no colonies visible to the naked eye) and from over culture media older than 2 days (with visible bacterial colonies) were examined. In addition, the air from empty chambers was tested. The measurement time was 20 min, including a 10-min testing exposure phase and a 10-min sensor regeneration phase. The results were analyzed in two variants: without baseline correction and with baseline correction. We tested 14 classifiers and found that a prototype of a multi-sensor recorder of the MCA-8 sensor signal was capable of detecting colonies of *P. l. larvae* on MYPGP substrate with a 97% efficiency and could distinguish between MYPGP substrates with 1–2 days of culture, and substrates with older cultures. The efficacy of copies of the prototypes M1 and M2 was shown to differ slightly. The weighted method with Canberra metrics (Canberra.811) and kNN with Canberra and Manhattan metrics (Canberra. 1nn and manhattan.1nn) proved to be the most effective classifiers.

## 1. Introduction

Semiconductor gas sensors (MOS) allow for the qualitative and quantitative identification of a gas or gas mixture due to the formation of a non-specific oxidation reaction of reducing gases adsorbed on the sensory layer. Then, on this layer, resistance changes occur, which can be converted into digital information expressed in volts [1,2].The MOS are low-cost and readily available, work in real-time, and provide an immediate score. Additionally, they show high sensitivity at the parts per million level. A set of semiconductor sensors was successfully applied as an electronic nose matrix that can recognize simple and complex odors [3,4].

The effectiveness of disease detection using an electronic nose has been scientifically proven many times. These devices are used in the diagnosis of plant [5], animal [6], and human [7] diseases. The most dangerous disease that plagues the honey bee is varroosis. It was shown that a matrix of six semiconductor gas sensors can distinguish bee colonies heavily infected with *Varroa destructor* from healthy ones [8], and this allows for the diagnosis of varroosis based on the examination of brood samples infected with this mite [9]. Another dangerous disease of bees is American foulbrood (AFB), caused by the *Paenibacillus larvae larvae* L. bacterium. This bacterium is a Gram-positive rod with dimensions of 0.5 and 1.5–6.0 m.

This bacterium produces endospores that are resistant to external environmental factors; therefore, its ability to infect may remain for over 30 years [10]. American foulbrood manifests in the dying out and rotting of bee larvae and pre-pupa in comb cells under the sealings. Consequently, this disease leads to the weakening of the colony and its death [11,12]. American foulbrood is recorded worldwide [13], and since it is considered very contagious, it is combated ex officio in most countries [14]. The symptoms of the disease are visible to the naked eye. Unfortunately, they are rarely noticed by the beekeeper in the early stages, and they are similar to the symptoms of other diseases affecting bee broods.

Therefore, laboratory diagnostics are essential. *P. l. larvae* in culturing on various media are colorless, transparent, and gray-white colonies; on agar with the addition of blood, they become mucoid non-hemolytic [15]. The appearance of bacterial colonies is not sufficient to identify the species of the pathogen that created them. Identification with labor-intensive chemical methods is necessary [15]. The primary structure of DNA allows for the distinction of five ERIC I–V genotypes, of which ERIC I and ERICII are most often found in infectious material [16].

In a rotting brood, a mixture of valeric, isocaproic, and caproic acids forms [17]. This results in a specific odor emitted by a bee colony with American foulbrood, which is perceptible even with a human nose [18]. Therefore, it seems reasonable to use an electronic nose for American foulbrood detection. We conducted field tests and analyzed the results. The high efficiency of a multi-sensor device in the detection of this disease in bee colonies was reported by a team of scientists from Australia [19].

The question we addressed in this study was whether an electronic nose can detect the presence of AFB-causing bacteria. The purpose of the experiment was, therefore, to determine to what extent and at what stage of multiplication of *P. l. larvae* on MYPGP agar (which contains yeast extract, Mueller-Hinton broth, glucose, K2HPO4, sodium pyruvate, and agar) the semiconductor sensors of volatile organic compounds can detect colonies of this bacterium.

## 2. Materials and Methods

The experiment was conducted at the Bee Products Quality Monitoring and Safety Laboratory of the Apiculture Division of the University of Warmia and Mazury in Olsztyn (Poland). A prototype of a multi-sensor recorder of the MCA-8 sensor signal with a matrix of six semiconductor gas sensors, created in the Laboratory of Sensor Technique and Indoor Air Quality Studies, Wroclaw University of Science and Technology, Poland, was tested. This device was created as part of the research project entitled “Development of innovative, intelligent tools monitoring the presence of American foulbrood and an increased level of *Varroa destructor* infestation in honeybee colonies”. Thus, by default, the task of this electronic nose is to monitor the most dangerous diseases affecting bees.

### 2.1. Construction and Mechanism of the MCA-8 Device’s Operation

The device uses semiconductor sensors from Figaro: TGS823, TGS826, TGS832, TGS2600, TGS2602, and TGS2603 (Table 1).

**Figure 1:**
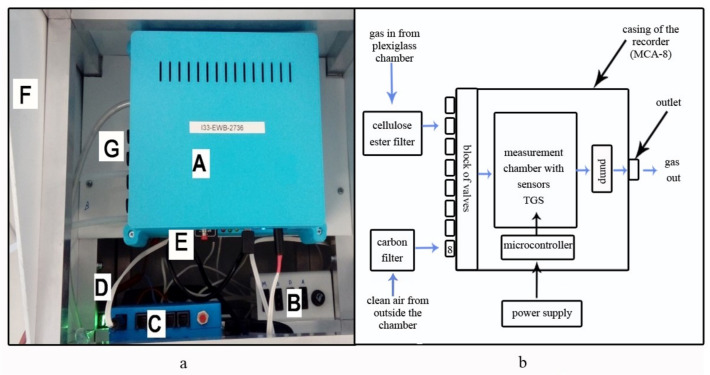
MCA-8 measurement device: (**a**) general view: A, multi-sensor recorder (MCA-8); B, power supply control panel; C, control panel for data transfer to the server; D, power cord; E, card reader; F, housing; and G, inlet channels; and (**b**) block diagram with marked construction elements and directional gas 80 flow.

MCA-8 has eight inlets and one outlet channel. The gas flow is forced by a software-controlled diaphragm pump. Each sensor is placed in its aluminum chamber so that the sample of the gas taken flows through all of them in sequence. This causes a voltage change on the surface of each sensor. In effect, measurement data arise that reflect a medium voltage registered on the sensor within 1 s before saving and sending the measurement result. The MCA-8 device can save data on the SD card and upload it to an external server (Figure 1).

### 2.2. Construction of the Measuring Stand

Two twin copies of the prototype of the MCA-8 measuring device were used in the study. The first device was named M1, and the second was named M2. Each prototype was attached with polyethylene (PE) tubes into the two test chambers with dimensions of 32×22×32 cm${}^{3}$. These tubes served as pre-sampling probes and provided a gas sample from the chamber to the MCA-8 prototype device through a specific channel number. Every two days, the channel number controlled by the software was changed, and then a tube was reattached to the next selected device channel.

**Figure 2:**
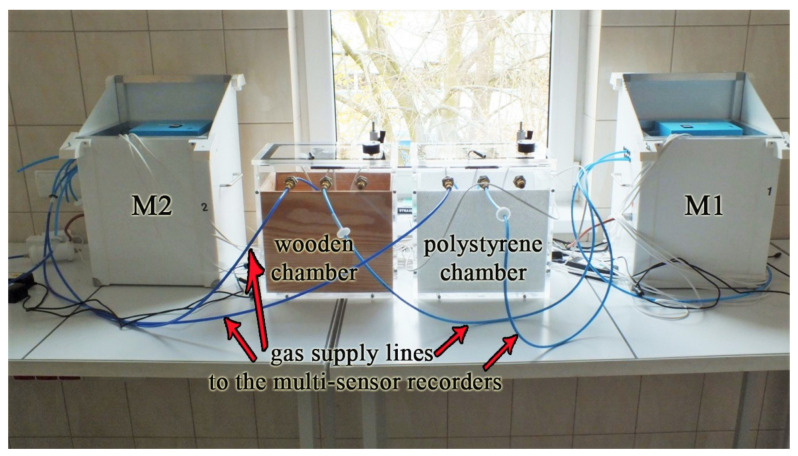
The measurement stand.

A filter composed of a mixture of cellulose ester (MCE 13 mm, 0.45 microns) was always located in the gas flow from the chamber to the device prototype. The chambers were composed of plexiglass. One chamber was lined with a wooden insert and the second with a polystyrene inserts. The inserts were used to reflect the conditions of the wooden and polystyrene hive because such materials are most often used in hives in beekeeping (Figure 2).

### 2.3. Preparation of Material for Research

**Figure 3:**
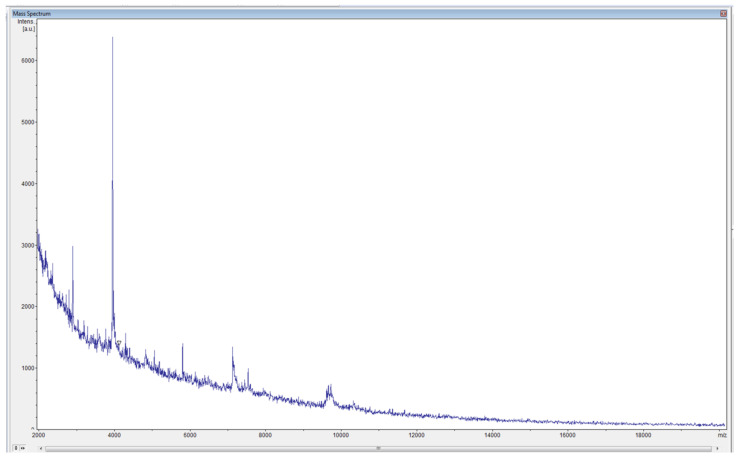
*P. l. larvae* (strain ATCC 9545, ERIC I): a spectrum of this bacterium obtained with a MALDI-TOF MS test.

The *P.l. larvae* ATCC 9545, ERIC I strain was used for the experiment. To identify the microbiological material obtained on the MYPGP media after culturing, MALDI-TOF-MS [20] testing was performed. The obtained spectrum confirmed that the examined bacterial colonies were the cultured strain of *P. l. larvae* ATCC 9545, ERIC I (Figure 3).

The bacteria culture was performed twice. In total, 64 Petri dishes with MYPGP media (which contains yeast extract, Mueller-Hinton broth, glucose, K2HPO4, sodium pyruvate, and agar) were used, with 32 plates used at once. After culturing, the plates were incubated in an incubator at 37 ${}^{\circ }$C. Placing the cultured plates in the incubator was considered day 0. Beginning the next day, on the first day of the experiment, three or four plates were selected daily from the incubator for the next ten days and they were examined with the MCA-8 device on the described laboratory stand.

**Figure 4:**
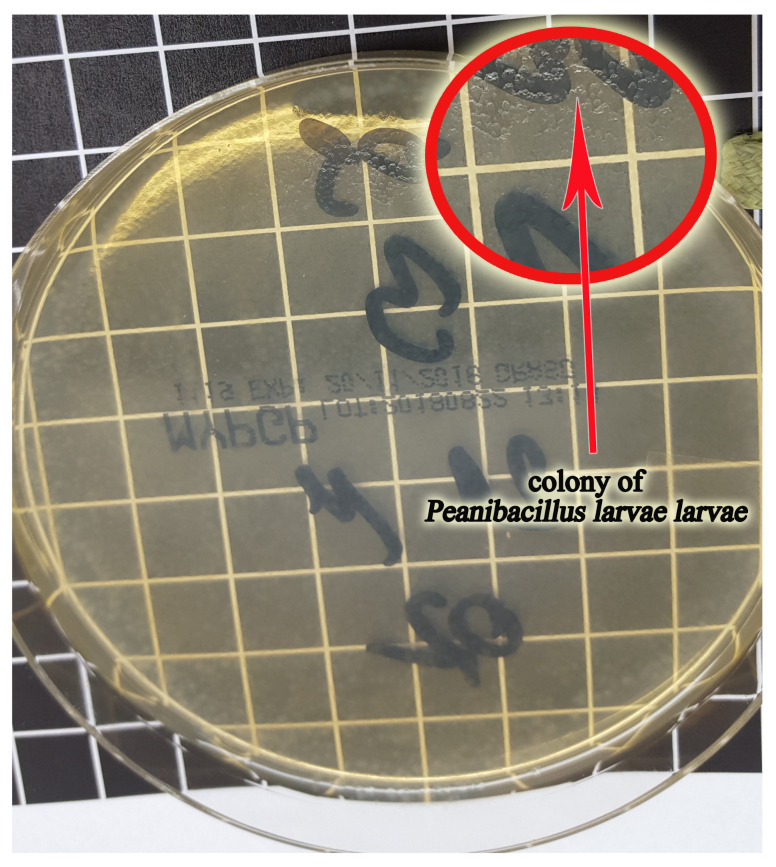
The Petri dish with MYPGP media (which contains yeast extract, Mueller-Hinton broth, glucose, K2HPO4, sodium pyruvate, and agar) with visible fine, transparent, and slimy colonies of the P.l.larvae; 6-day culture.

No bacterial colonies were observed on plates with for the first 2 days of culture. Colonies visible to the naked eye already developed on the plates with the 3 day culture. The colonies were 1–3 mm in diameter; with a smooth, shiny, and slimy consistency; and regular shape. Due to their colorlessness, they appeared transparent *P.l. larvae* (Figure 4).

### 2.4. Decision Classes

We tested gas samples from above the media with the culture of one- and two-day-old *P. l. larvae* (class 23) and with visible bacterial colonies (cultures older than 2 days (class 24)). High-class numbers indicate laboratory research covering many different objects concerning living in a hive environment. The lower-class numbers were reserved for other objects. Air from an empty test chamber (class 1) was used as a control. Table 2 shows how many objects were located in a specific attribute and variant.

### 2.5. *Measurement Process*

**Figure 5:**
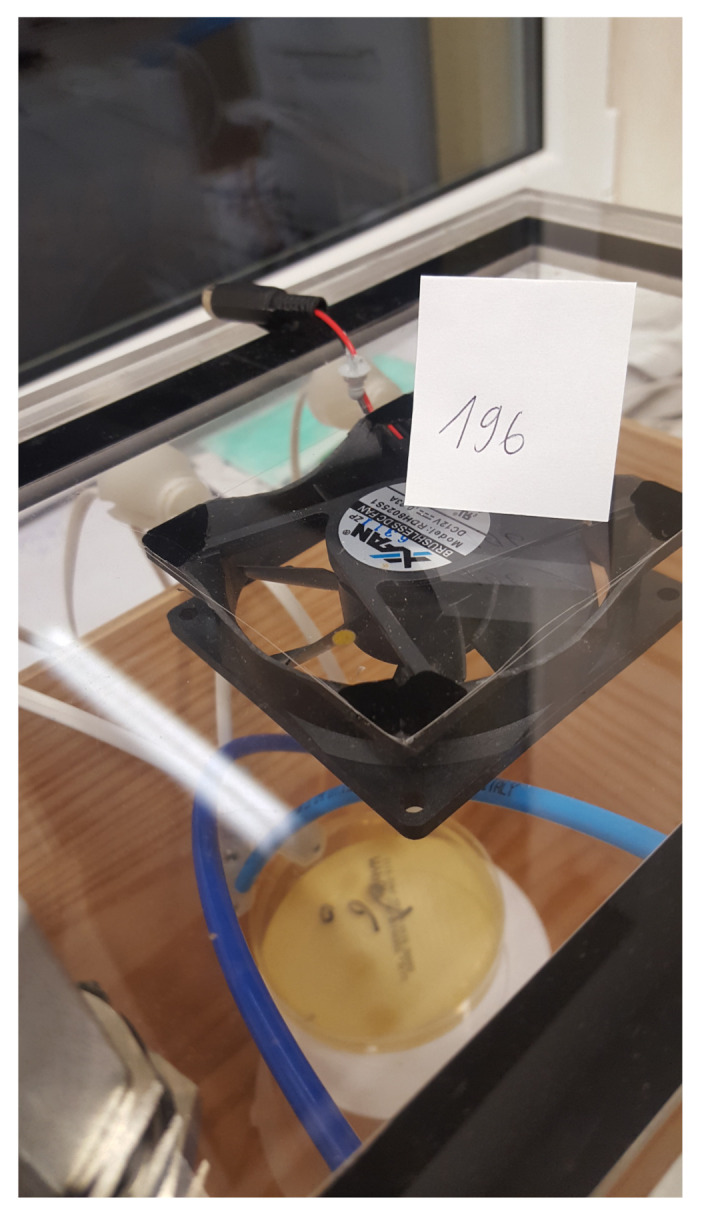
Measurement of a Petri dish with MYPGP media without visible colonies (class 23) in a wooden chamber.

For classes 23 and 24, the examination of the sample consisted of placing a Petri dish with the contents in a wooden or polystyrene chamber under the outlets of two pipes sucking in gas to both MCA-8 devices (Figure 5).

**Figure 6:**
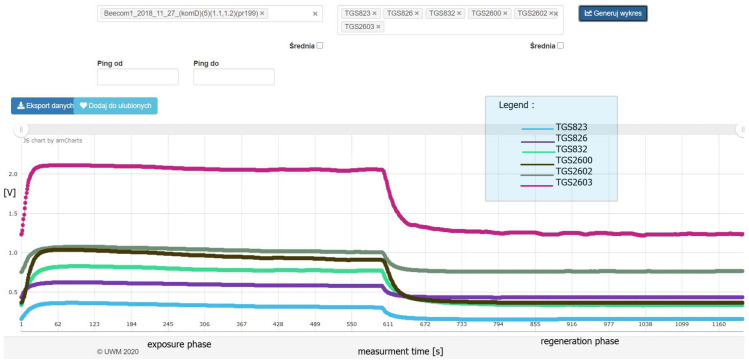
A sample graph illustrating the process of measurement. In this case, the measurement concerned a colony of *P. l. larvae* 6 days after culture in a wooden chamber.

Both devices were set simultaneously for 10 min. This time was called the exposure phase. Then, the device entered a regeneration phase for 10 min. This phase consisted of suction of clean air through channel 8 from outside the chamber through a carbon filter. In the case of class 1, the procedure was the same, only the chambers were empty. A single measurement resulted in 1200 sensor readings registered every second, of which the first 600 readings corresponded to the exposure phase and the other 600 to the regeneration phase. As shown in the example in Figure 6, the sensor readings stabilized after the first minute of device operation. The tests were carried out at 21 ${}^{\circ }$C.

### 2.6. The Analyzed Variable

The analyzed variable was the response of the sensor at the selected time point of sample exposure to differential baseline corrections. Therefore, the following were selected for analysis: the value from 270 s of sensor readings from the measurement of a sample with baseline correction by subtracting these reading values from the last 600 s of the surrounding air measurement.

### 2.7. Visualization of the Tested Classes

First, the obtained mean sensor readings were visualized using radial charts. As such, images of the sensor matrix were obtained for individual devices and classes regarding both variants. To achieve our goal, we normalized the values of the TGS attributes to a range of [0, 1] using Equation (1). Normalization of $TGS_{i}\left(ob_{j}\right)$ (the *i*th descriptor of the *j*th object into the [a,b] interval) consisted of the following step:

(1) $$TGS_{i}\left(ob_{j}\right)=\frac{\left(TGS_{i}\left(ob_{j}\right)-min_{TGS_{i}}\right)*\left(b-a\right)}{max_{TGS_{i}}-min_{TGS_{i}}}+a.$$

Then, we calculated the average values in the classes for the individual TGS attributes—see Equation (2)—and squared the obtained values using Equation (3), which means that a step was being taken.

(2) $$average\left(TGS_{i}^{class_{j}}\right)=\sum_{k=0}^{|class_{j}|}TGS_{j}\left(ob_{l}\right),\;where\;ob_{l}\in class_{j}$$

(3) $$average\left(TGS_{i}^{class_{j}}\right)=average{\left(TGS_{i}^{class_{j}}\right)}^{2}.$$

#### 2.7.1. Image of the Sensors Matrix of the M1 Device

**Figure 7:**
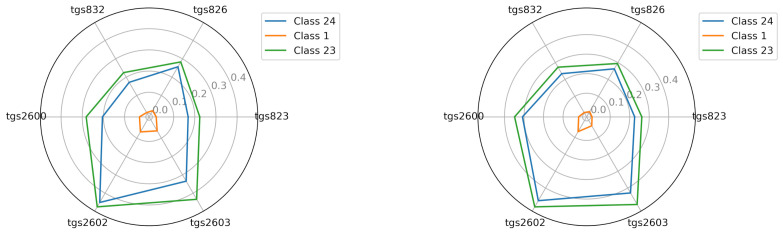
The visualization of the average reading intensity of TGS sensors for decision classes 1, 23, and 24 using M1; wooden chamber on the left and polystyrene chamber on the right.

Through visualization of the average reading intensity of the TGS sensors, an image of the sensors matrix was obtained. With the M1 device, the lowest readings were obtained by the sensors when measuring the empty chamber. All TGSs obtained average values did not exceed 0.1 V. Higher average sensor readings were observed for class 23 than for class 24. The highest values, both for the wooden chamber and the polystyrene chamber, were obtained from sensors TGS2602 and TGS2603, which were close to 0.45 V (Figure 7).

#### 2.7.2. Image of the Sensor Matrix of the M2 Device

**Figure 8:**
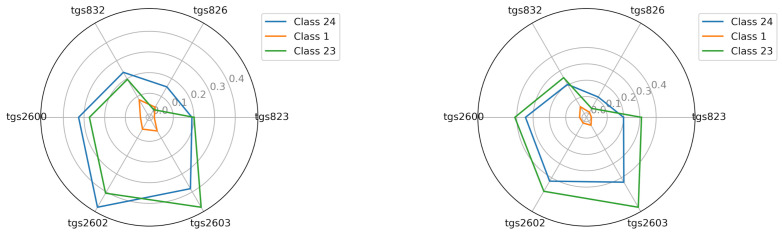
The visualization of the average reading intensity of TGS sensors for decision classes 1, 23, and 24 using M2; wooden chamber on the left and polystyrene chamber on the right.

The M2 device in a wooden chamber produced average readings in four cases of sensors for class 23 that were lower than for class 24. TGS2603 in class 23 was more sensitive (above 0.45 V) than in class 24 (below 0.4 V) (Figure 8).

The low sensitivity showed by the device was due to the M2 sensor TGS826, which, in both types, produced class 23 readings similar to those for class 1 (0.05 V). In class 24 for all cases, this sensor produced a higher average reading because it fit between 0.1 and 0.15 V (Figure 8); however, it was much less than those produced by the M1 device on M1, where for both chambers, the readings fit between 0.25 and 0.3 V (Figure 7). The average readings of other sensors for M2 device were higher and more similar to the readings obtained by the M1 device.

### 2.8. Brief Approach to the Methods

The aim of this work was to construct a simple and effective classification technique to address the problem under investigation. To achieve this goal, we needed to design a classifier for a numeric decision system with small unbalanced decision classes that characterized the data being studied. We decided to choose the most natural problem-solving techniques, including the k nearest neighbour method (see [21]), the naïve Bayes method (see [21]), and our own weighted voting technique Algorithm 811 (see [21]).

The research was conducted using the cross-industry standard process for data mining (CRISP-DM) methodology. We used a range of metrics in these methods. Studies with dedicated methods were conducted according to the Monte Carlo cross-validation (MCCV) model, using the same subsets of data for all techniques studied. To correctly estimate the effectiveness of unbalanced data, we used balanced accuracy (the equivalent of the recall parameter) and the true positive rate (the equivalent of the precision parameter). Where more than two classes were classified, each of these parameters was calculated separately for each class.

Balanced accuracy is the average accuracy of the classification from all decision classes, whereas the accuracy of classification of a given test class is understood as the percentage of correctly classified objects in that class. The true positive rate is the percentage of objects of a given class positively classified in relation to all objects classified in that class. The coverage parameter is the percentage of classified objects. In our case, it was always equal to one; in other words, each test object was classified.

Next, we provide a more detailed discussion of the methodology used.

#### Summary of the Classifiers

The options we tested were canberra.1nn, canberra.2nn, canberra.3nn, canberra.811, eps = 0.01.nb, euclidean.1nn, euclidean.2nn, euclidean.3nn, euclidean.811, manhattan.1nn, manhattan.2nn, manhattan.3nn, manhattan.811, and nb.num. In this subsection, we discuss the general outline of each of these options. We provide a detailed description of our kNN classifier variant (canberra.1nn, canberra.2nn, canberra.3nn, euclidean.1nn, euclidean.2nn, euclidean.3nn, manhattan.1nn, manhattan.2nn, and manhattan.3nn).

Classification involves voting for the k nearest objects from each decision class. Consider the training decision system $\left(U^{trn},TGS,class\right)$ and the test decision system $\left(U_{tst},TGS,class\right)$ as inputs, where TGS is a set of conditional attributes and class is the decision attribute. The classification of test objects using the training objects was performed as follows: For all conditional attributes tgs∈TGS, training objects $v\in U^{trn}$, and test objects $u\in U_{tst}$, we computed weights w(u,v) based on the selected metric. The metrics used in the kNN method are shown in Table 3.

Classification was based on the summed weight of the k nearest objects of each decision class.

For a general perspective of the Bayes classifier, please see cf., Mitchell [22], Devroye et al. [23], and Duda et al. [24]. The naïve Bayes classifier owes its naivety epithet to one assuming the independence of attributes; in reality, this condition is not often met. Its working in the realm of decision systems can be described concisely as follows: For a given training decision system $\left(U_{trn},TGS,class\right)$ and a test system $\left(U_{tst},TGS,class\right)$, where $U=U_{trn}\cup U_{tst}$ is the set of objects, $TGS=\{tgs_{1},tgs_{2},\ldots ,tgs_{n}\}$ is the conditional attribute set, and $class\in CLASS=\{class_{1},class_{2},\ldots ,class_{k}\}$ is the decision attribute. The classification of a test object $v\in U_{tst}$, described using its information set $\left(tgs_{1}\left(v\right),tgs_{2}\left(v\right),\ldots ,tgs_{n}\left(v\right)\right)$, consists of computing, for all decision classes, the value of the parameter

$$P(class=class_{i}|b_{1}=tgs_{1}\left(v\right),b_{2}=tgs_{2}\left(v\right),\ldots ,b_{n}=tgs_{n}\left(v\right)),$$

and the decision on *v* is the decision value with the maximal value of the parameter. In practice, we can use partial estimation

$$P(b_{m}=tgs_{m}\left(v\right)|class=class_{i})=$$

$$\frac{\mathrm{number}\;\mathrm{of}\;\mathrm{test}\;\mathrm{instances}\;b_{m}=tgs_{m}\left(v\right)\;\mathrm{in}\;\mathrm{training}\;\mathrm{class}\;class_{i}}{\mathrm{cardinality}\;\mathrm{of}\;\mathrm{class}\;class_{i}}.$$

Each decision class is voted by submitting the value of the parameter

(4) $$Param_{class=class_{i}}=P\left(class=class_{i}\right)*\sum_{m=1}^{n}P\left(b_{m}=tgs_{m}\left(v\right)|class=class_{i}\right).$$

This classifier was fit for symbolic attributes. The method was used on numerical data in variant (eps = 0.01.nb) by applying the descriptors’ indiscernibility degree (eps parameter).

The last algorithm 811 is based on the weighted voting idea. In variants canberra.811, euclidean.811, and manhattan.811, we used the appropriate metrics (Table 3) Considering the distance between objects as

$$w\left(u,v\right)=\sum_{i=1}^{6}]\frac{|TGS_{i}\left(u\right)-TGS_{i}\left(v\right)|}{max_{training\;set}TGS_{i}-min_{training\;set}TGS_{i}}.$$

$max_{training\;set}TGS_{i}$ and $min_{training\;set}TGS_{i}$ are the largest and smallest values, respectively, of the given conditional attribute. Classification by this method consists of calculating the following parameter for each decision class of the training system:

$$Param_{class_{j}}=\frac{\sum w\left(u,v\right),v\in class_{j}}{|class_{j}|}.$$

## 3. A Few General Statements about the Experiments

In the following sections, we present the results of our research into the classification of classes 1, 23, and 24 in the context of a multi-class problem, and the binary classification using the one vs. one (one class vs. another class) and one vs. other (one class vs. other classes) strategy. We considered the test options mentioned above:

- M1 device in a wooden chamber, and
- M1 device in a polystyrene chamber,

as well as similar options for the M2 device.

## 4. Results for the Configuration One vs. Other

### 4.1. Analysis of the Results of the 5 × MCCV-5 Validation Tests in the Comparison: Specific one vs. Other (Class vs. other Classes): Choosing the Most Effective Classification Method

The highest values for acc_class_, tpr_class_, and acc_balanced_ obtained in tests in the comparison class vs. other classes as well the classifiers that enabled obtaining these values are presented in Table 4, Table 5 and Table 6.

The canberra.811 test was the most effective classifier, providing the highest results in terms of acc_class_, tpr_class_, and acc_balanced_ parameters in 11 cases out of 41. The following tests also performed well: canberra.1nn and euclidean.811 (seven cases with the highest parameters each). However, the nb.num test never provided the highest value (Table 7).

### 4.2. Results for the Configuration One vs. Other, canberra.811

With the canberra.811 method, class 1, i.e., empty chamber, was well-recognized by both devices, where most parameter values for the canberra.811 test were above 0.7. In both cases, tpr1 was lower: for M1, it was 0.661 for the wooden chamber; and for M2, it was 0.590 for the polystyrene chamber (Table 8).

For class 23 (MYPGP media with cultured bacteria but with no visible colonies), the results of canberra.811 were not satisfactory. For the two devices, a very low tpr_23_ was obtained: 0.2 and lower (Table 8).

For class 24, high acc_24_, tpr_24_, and acc_balanced_ values of 0.7 or higher were obtained by both M1 and M2 devices. Only acc_balanced_ for M2 polystyrene chamber was lower at 0.681 (Table 8), but this value is satisfactory.

## 5. Results of One vs. One

### 5.1. Results for the Configuration 23 vs. 24, manhattan.1nn

In classification with model 5 × MCCV-5 for classes 23 and 24, the tpr24 was the most satisfactory with the manhattan.1nn method (Table 9). Observing the results of the manhattan.1nn method in configuration 23 vs. 24, we found that the M1 device in a polystyrene chamber produced satisfactory separability of both classes. In the wooden chamber, a very low tpr_23_ was obtained, accompanied by an acc_balanced_ below 0.6. In the case of the M2 device, we concluded that both classes were perfectly separable, and the M2 device achieved an excellent distinction between the MYPGP media with the culture of *P. l. larvae* without colonies and the MYPGP media with colonies of this bacteria (Table 9).

### 5.2. Results for the Configuration 1 vs. 23, canberra.811

The results of the analyses in configuration 1 vs. 23 were not indicative of the best classifier, as many methods obtained excellent separability parameters for both classes. The effectiveness of the canberra.811 method is demonstrated in Table 10. The separability of both classes was at a high level and ranged from 85% for device M1 in a wooden chamber to 100% for device M2 in a polystyrene chamber (Table 10).

### 5.3. Results for the Configuration 1 vs. 24, canberra.1nn

The comparison of class 1 data vs. class 24 data in the 5 × MCCV-5 validation tests provided satisfactory separability results for both classes. Most of the classifiers worked perfectly with all variants. Table 11 shows the results of tpr_class_ and acc_balanced_ for the canberra.1nn method. From the results, the tpr_class_ in all variants was very high, always over 0.9 (Table 11).

### 5.4. Discussion

Prompt classification of pathogenic microorganisms is essential for effective disease treatment. To date, numerous complicated and time-consuming methods have been used in microbiological diagnostics [25,26]. Due to the complexity of metabolic processes, bacteria produce a series of volatile compounds (VCs) [27,28,29,30,31]. Therefore, the use of an e-nose based on gas sensors appears to be a potential method that overcomes these drawbacks [32,33].

The amount and composition of volatile compounds depend on both the medium on which the bacteria develop [34] and the species of the microorganism [35,36]. The detection of pathogenic bacteria and their identification with an e-nose can be successfully achieved in both the infected in organisms (in vivo) [33] and in laboratories (in vitro) [32].

Different types of sensors may be applied here. The performance of coated porphyrin and quartz sensors with a gas microbalance was analyzed by [37], who found that these sensors can distinguish the VCs produced by 12 microorganisms in vitro, as well as being able to classify the bacteria as Gram-positive or negative. An electronic nose, Cyranose 320, based on thirty-two polymer carbon black composite sensors can detect six species of bacteria causing eye infections with 98% efficiency [38].

Semiconductor gas sensors (MOS) have also been used in the detection and identification of bacteria [33]; by examining the bacterial biofilm on dental plaque with a matrix of six sensors of the TGS series, these sensors were able to recognize teeth and mouth diseases [32]. An e-nose based on a matrix of 28 semiconductor sensors was able to recognize pneumonia in people infected with *Pseudomonas aeruginosa* with 92% efficiency. *Listeria monocytogenes* and *Bacillus cereus* bacteria incubated in Tryptic soy broth (TSB) were identified by a set of metal oxide sensors with 98% efficiency [35].

*Staphylococcus carnosus, Staphylococcus xylosus, Staphylococcus saprophyticus, Staphylococcus warneri, Staphylococcus epidermidis, Staphylococcus aureus*, and *Micrococcus varians* were classified by an e-nose with a six-sensor TGSXXX matrix with 90.5% efficiency. The satisfactory results in identifying microbes that were obtained by other researchers indicate the research direction related to MCA-8 device testing. As this e-nose is intended to be used for the diagnosis of bee diseases in the future, it should also be proven in the detection of *P. l. larvae* bacteria that are more dangerous to bee brood. The most frequently isolated infected larvae ERIC I strain was selected for this research [39].

Many researchers, when verifying the effectiveness of e-noses in detecting bacteria, relied on one device only. In our experiment, gas from above the tested object was directed into two twin MCA-8 devices specified as M1 and M2 simultaneously. This enabled the comparison of their sensor matrix images as well as their effectiveness. Even though each MCA-8 device had a set of the same sensors, we could immediately observe that the images from each matrix appeared different. Visual comparison of mean readings for individual TGS sensors in individual classes using radial graphs showed that classes are distinguishable within the M1 and M2 devices, but they do not indicate a general rule applicable to both devices.

Thus, M1 produces a different image of the sensor matrix than M2 on the tested gas. These results of our experiment confirm there are no identical sensors with repeatable readings. This problem is called sensor drift [40] and each device requires an individual approach to calibration, for example, by using an appropriate algorithm [41].

The images of the matrix of the sensor readings obtained for the wooden and polystyrene chambers were visually similar. The raw data used for the analyses were subjected to differential baseline correction. Baseline correction has been recommended by many researchers [42,43,44,45]. Improvement in classification efficiency is the aim of such a correction. It is especially justified under changing environmental conditions. In our case, the experiment was conducted under stable laboratory conditions. However, in the final analyses, applying a baseline correction improved the results.

The variant of the multi-class classification in the MCCV-5 model does not provide a clear answer as to which method can guarantee the most accurate classification of the tested objects. However, class 23 was the hardest to separate; in most cases, it was not possible at all. Classes 1 and 24 were easier to separate.

In the next step, the results of the 5 × MCCV-5 validation classification in the one vs. other (specific class vs. other classes) configuration were validated. The summary of the highest parameter values of class separability obtained by specific classifiers allowed us to select the canberra.811method as the best. However, it did not always produce satisfying results in our experiements. A very low tpr_23_, below 0.3 for the M1 device was observed.

In connection with the unfavorable separability of class 23, the results of 5 × MCCV-5 validation in the one vs. one configuration (class vs. other class) were also examined. First, the data of class 23 vs. class 24 were checked. We used 15 classifiers, and the manhattan.1nn classifier was found to be the most accurate. With this classifier, the M1 device in the wooden chamber could not detect the MYPGP media with culture but with no colonies (accuracy level 14%), and in the polystyrene chamber, the detection level was very low (42%) (Table 9). In contrast, the M1 device was excellent at detecting *P. l. larvae* colonies on the MYPGP media. In the wooden chamber, the effectiveness was 0.81%, and in the polystyrene chamber, 0.85%.

The M2 device in the configuration of classes (23 vs. 24) was more accurate because it was effective in detecting both the first and the second class. The detection over MYPGP media with no bacteria colonies in the wooden chamber was 66% effective, and in the polystyrene, it was 70% effective. The *P. l. larvae* bacteria colonies in the both chambers were recognized by the M2 device with an efficiency of 88% (Table 9).

Therefore, we identified the problem of the lack of class 23 separability in the polystyrene chamber with the M1 device; we then decided to compare data of this class vs. class 1 data. The 5 × MCCV-5 validation results in this class configuration resulted in perfect class 23 separability. Such effects were obtained by most of the classifiers. Based on the canberra.811 method, we found that the M1 device detected class-assigned objects with over 85% efficiency, and the M2 device, with an accuracy of 100% in almost every case (Table 10).

To complete the analyses, a study was performed in the configuration of class 1 vs. class 24. With the use of superior classifiers, the 5 × MCCV-5 validation results were favorable. Using the example of the canberra.1nn method, we observed that the M1 device could recognize an empty wooden chamber with an accuracy of 81%, and a polystyrene one with an accuracy of 100%. The recognition of the *P. l. larvae* bacterial colony through this device remained at the level of 91% in the wooden chamber and 100% in the polystyrene chamber (Table 11).

The M2 device was even more effective in detecting an empty chamber. The result for the wooden chamber was 97% and 100% for polystyrene. Colonies of *P. l. larvae* bacteria were recognized by the M2 device in the wooden chamber with 99% efficiency, and in a polystyrene chamber with 96% efficiency (Table 11).

The experiment, therefore, showed that *P. l. larvae* colonies on the MYPGP medium in laboratory conditions can be easily detected by our MCA-8 prototype of a multi-sensor recorder of sensor signals. We can expect average effectiveness of more than 97%. Comparing the results obtained in the in vitro detection of bacteria by other researchers [35,36], who tested e-noses based on the same sensors as MCA-8, the results of the experiment can be considered excellent. It is clear, that the laboratory results presented here are for a highly simplified laboratory model. Therefore, our team also tested three specimens of the MCA-8 device under live bee colony conditions. The results and conclusions from these tests will be presented in the next publication. The multi-sensor recorder used in our experiment in earlier studies perfectly detected bee broods strongly infected with the dangerous parasitic mite *Varroa destructor*. This indicates that our e-nose has considerable potential to be applied in the detection of bacteria that are dangerous to bees and, therefore, the diseases caused by them.

## 6. Conclusions

(1) The *P.l. larvae* colonies on MYPGP media were detected by the MCA-8 prototype of a multi-sensor recorder of the sensor with 97% efficiency.
(2) Objects, such as an empty wooden chamber, an empty polystyrene chamber, MYPGP media with 1–2 days’ bacteria culture (no visible colonies), and MYPGP media with visible *P.l. larvae* colonies were distinguished by the MCA-8 device.
(3) The M1 unit of the MCA-8 device was slightly less effective in object detection than the M2 unit.
(4) A different average image obtained by the matrices of the sensors used was generated by each unit of a prototype of a multi-sensor recorder of the MCA-8 sensor signal.
(5) The environment of the tested object did not significantly influence the effectiveness of the MCA-8 device. The results of the recognizability of the examined objects were satisfactory and comparable for both the wooden and polystyrene chambers.
(6) The most effective classifiers proved to be: weighted method with Canberra metric (canberra.811) and kNN with the Canberra and Manhattan metric (canberra. 1nn and manhattan.1nn).
(7) When applying the 5 × MCCV-5 quality classification model, the best results were obtained in the one vs. one binary classification variant.

## Figures and Tables

**Table 1:** The characteristics of semiconductor gas sensors, which were used in the multi-sensor array ([9], https://www.figaro.co.jp/en (accessed on 17 July 2021)).

Sensor	SubstancesDetected	DetectionRange
TGS823	Organicsolventvapors	50∼5000 ppm, *ethanol, n-hexane, benzene, acetone*
TGS826	Ammonia	30∼300 ppm, ethanol, ammonia, isobutane
TGS832	Chlorofluorocarbons	100∼3000 ppm, R-407c, R-134a, R-410a, R-404a, *R-22*
TGS2600	Gaseousaircontaminants	1∼100 ppm
TGS2602	VOCsandodorousgases	1∼30 ppm, ethanol, ammonia, toluene
TGS2603	Amine-seriesandsulfurousodorgases	1–30 ppm, ethanol, 0.1–3 ppm trimethylamine, 0.3–2 ppm methyl mercaptan

**Table 2:** Size of decision classes in the tested variants.

Variant/ClassName	1	23	24
M1woodenchamber	14	12	49
M1polystyrenechamber	14	11	49
M2woodenchamber	9	11	50
M2polystyrenechamber	9	11	49

**Table 3:** Metrics used in the kNN method.

Canberra metric	$w\left(u,v\right)=\sum_{i=1}^{6}\frac{|TGS_{i}\left(u\right)-TGS_{i}\left(v\right)|}{|TGS_{i}\left(u\right)|+|TGS_{i}\left(v\right)|}$
Euclidean metric	$w\left(u,v\right)=\sqrt{(}\sum_{i=1}^{6}{\left(|TGS_{i}\left(u\right)-TGS_{i}\left(v\right)\right)}^{2})$
Manhattan metric	$w\left(u,v\right)=\sum_{i=1}^{6}\left|TGS_{i}\left(u\right)-TGS_{i}\left(v\right)\right|$

**Table 4:** Summary of the highest acc_class_ values obtained in the 5 × MCCV-5 validation tests: specific class vs. other classes including the classifiers that enabled obtaining them: devices M1 and M2.

Parameter	acc_class_			
**Device**	**M1**		**M2**	
**Chamber**	**Wooden**	**Polystyrene**	**Wooden**	**Polystyrene**
Configuration	1 vs. other classes			
Max	0.845	1.000	1.000	1.000
The best method	canberra.811	euclidean.811	euclidean.811	euclidean.811
		manhattan.811	manhattan.811	manhattan.811
Configuration	23 vs. other classes			
Max	0.697	0.887	0.970	0.983
The best method	canberra.811	canberra.811	euclidean.811	manhattan.811
Configuration	24 vs. other classes			
Max	0.933	0.994	0.847	0.864
The best method	euclidean.3nn	canberra.811	canberra.811	canberra.1nn

**Table 5:** Summary of the highest values of tpr_class_ obtained in the 5 × MCCV-5 validation tests: specific class vs. other classes including classifiers that enabled obtaining the result (the best method) for devices M1 and M2.

Parameter	tpr_class_			
**Device**	**M1**		**M2**	
**Chamber**	**Wooden**	**Polystyrene**	**Wooden**	**Polystyrene**
configuration	1 vs. other classes			
max	0.800	0.937	0.993	0.977
the best method	euclidean.1nn	canberra.2nn	canberra.1nn	canberra.1nn
			canberra.2nn	
configuration	23 vs. other classes			
max	0.233	0.485	0.735	0.608
the best method	euclidean.2nn	euclidean.2nn	canberra.1nn	euclidean.1nn
configuration	24 vs. other classes			
max	0.786	0.851	0.869	0.848
the best method	canberra.811	canberra.1nn	euclidean.811	euclidean.1nn

**Table 6:** Summary of the highest acc_balanced_ values obtained in the 5 × MCCV-5 validation tests: specific class vs. other classes including classifiers that enabled obtaining these values (the best method) for devices M1 and M2.

Parameter	acc_balanced_			
**Device**	**M1**		**M2**	
**Chamber**	**Wooden**	**Polystyrene**	**Wooden**	**Polystyrene**
configuration	1 vs. all			
max	0.865	0.792	0.972	0.983
the best method	canberra.811	canberra.3nn	canberra.1nn	euclidean.1nn
		euclidean.811		
configuration	23 vs. all			
max	0.519	0.651	0.645	0.648
the best method	manhattan.2nn	euclidean.811	canberra.811	euclidean.1nn
configuration	24 vs. all			
max	0.717	0.801	0.700	0.681
the best method	canberra.811	canberra.1nn	canberra.811	canberra.811

**Table 7:** The effectiveness of individual classifiers expressed as a number indicating how many times a specific test produced the highest value of acc_klass_, tpr_class_, or acc_balanced_ in all comparisons of class vs. other.

Method	${\mathrm{acc}}_{\mathrm{class}}$		${\mathrm{tpr}}_{\mathrm{class}}$		${\mathrm{acc}}_{\mathrm{balanced}}$		Total
	M1	M2	M1	M2	M1	M2	
canberra.1nn	0	1	1	3	1	1	7
canberra.2nn	0	0	1	1	0	0	2
canberra.3nn	0	0	0	0	1	0	1
canberra.811	4	1	1	0	2	3	11
eps=0.01.nb	0	0	0	0	0	0	0
euclidean.1nn	0	0	1	2	0	2	5
euclidean.2nn	0	0	2	0	0	0	2
euclidean.3nn	1	0	0	0	0	0	1
euclidean.811	1	3	0	1	2	0	7
manhattan.1nn	0	0	0	0	0	0	0
manhattan.2nn	0	0	0	0	1	0	1
manhattan.3nn	0	0	0	0	0	0	0
manhattan.811	1	3	0	0	0	0	4
nb.num	0	0	0	0	0	0	0
sum	7	8	6	7	7	6	41

**Table 8:** The obtained acc_class_ values in the 5 × MCCV-5 validation tests in the comparison of specified class vs. other classes for the test canberra.811 for devices M1 and M2.

Method	canberra.811			
**Device**	**M1**		**M2**	
**Chamber**	**Wooden**	**Polystyrene**	**Wooden**	**Polystyrene**
configuration	1 vs. all			
acc_1_	0.845	0.988	0.978	0.971
tpr_1_	0.661	0.828	0.701	0.590
acc_balanced_	0.864	0.968	0.947	0.936
configuration	23 vs. all			
acc_23_	0.697	0.876	0.970	0.992
tpr_23_	0.152	0.167	0.225	0.202
acc_balanced_	0.519	0.616	0.645	0.604
configuration	24 vs. all			
acc_24_	0.902	0.994	0.847	0.864
tpr_24_	0.786	0.815	0.831	0.789
acc_balanced_	0.717	0.784	0.701	0.681

**Table 9:** The obtained tpr values for class 23 and 24, and acc_balanced_ in validation tests of 5 × MCCV-5 in comparison of 23 vs.24 for manhattan.1nn test, devices M1 and M2.

Configuration	23 vs. 24			
**Method**	manhattan.1nn			
**Device**	**M1**		**M2**	
**Chamber**	**Wooden**	**Polystyrene**	**Wooden**	**Polystyrene**
$tpr_{23}$	0.138	0.423	0.657	0.702
$tpr_{24}$	0.806	0.849	0.877	0.880
$acc_{balanced}$	0.450	0.596	0.676	0.695

**Table 10:** The obtained tpr values for class 1 and 23, and acc_balanced_ in validation tests of 5 × MCCV-5 in the comparison of 1 vs.23 for the canberra.811 test, device M1 and M2.

Configuration	1 vs. 23			
**Method**	manhattan.1nn			
**Device**	**M1**		**M2**	
**Chamber**	**Wooden**	**Polystyrene**	**Wooden**	**Polystyrene**
$tpr_{23}$	0.853	0.910	1.000	1.000
$tpr_{24}$	0.870	1.000	0.981	1.000
$acc_{balanced}$	0.851	0.987	0.987	1.000

**Table 11:** The obtained tpr values for classes 1 and 24, and acc_balanced_ in the validation tests of 5 × MCCV-5 in the comparison of 1 vs. 24 for the canberra.1nn test, devices M1 and M2.

Configuration	1 vs. 24			
**Method**	canberra.1nn			
**Device**	**M1**		**M2**	
**Chamber**	**Wooden**	**Polystyrene**	**Wooden**	**Polystyrene**
$tpr_{1}$	0.913	1.000	0.973	1.00
$tpr_{24}$	0.914	0.997	0.992	0.958
$acc_{balanced}$	0.826	0.996	0.977	0.894
